# Mesotherapy With Lidocaine and Ketamine in Complex Regional Pain Syndrome Type 1: A Randomized Double‐Blind Trial

**DOI:** 10.1111/papr.70187

**Published:** 2026-07-08

**Authors:** Philippe Lafuma, Laurent Magaud, Amna Abichou‐Klich, Muriel Rabilloud, Julien Wegrzyn, Christian Baude, Pascal Rippert, Myriam Kaou, Julie Haesebaert, Frédéric Rongieras, Chloé Marchand, Carole Dhelens, Olivia Febvey‐Combes

**Affiliations:** ^1^ Hospices Civils de Lyon Groupement Hospitalier Centre Edouard Herriot, Service de Chirurgie Orthopédique et Urgences Traumatologiques du Membre Inférieur Lyon France; ^2^ Hospices Civils de Lyon Pôle de Santé Publique, Service Recherche et Epidémiologie Cliniques Lyon France; ^3^ Hospices Civils de Lyon Service de Biostatistique‐Bioinformatique Lyon France; ^4^ Université Claude Bernard Lyon 1, UMR 5558, CNRS Villeurbanne France; ^5^ Service D'orthopédie et Traumatologie Centre Hospitalier Universitaire Vaudois Lausanne Switzerland; ^6^ Centre de la Douleur Médipôle Lyon Villeurbanne Villeurbanne France; ^7^ Hospices Civils de Lyon Pôle de Santé Publique, Unité Bases Cliniques et Epidémiologiques Lyon France; ^8^ Research on Healthcare Performance RESHAPE, INSERM U1290, Université Claude Bernard Lyon 1 Lyon France; ^9^ Hospices Civils de Lyon Groupement Hospitalier Centre Edouard Herriot, Pharmacie, FRIPHARM Lyon France

**Keywords:** complex regional pain syndrome, ketamine, lidocaine, mesotherapy, randomized trial

## Abstract

**Background:**

Complex regional pain syndrome type 1 (CRPS1) is a disabling pain syndrome whose treatment remains challenging. Mesotherapy is a therapeutic method involving the injection of low doses of active substances through the skin. No studies assessed mesotherapy in CRPS1 patients. The aim of this study was to evaluate the efficacy on pain relief of ketamine and lidocaine mixtures versus lidocaine alone administered by mesotherapy.

**Methods:**

This three‐arm, randomized, double‐blind trial compared mesotherapy with lidocaine 20 mg alone or mixed with ketamine at a dose of 20 mg or 40 mg, in addition to standard treatment. The primary outcome was the difference in pain between inclusion and D56 after four mesotherapy sessions, assessed by a visual analog scale (VAS).

**Results:**

Thirty‐one patients were randomized to receive lidocaine 20 mg (*n* = 10), lidocaine 20 mg + ketamine 20 mg (*n* = 11), or lidocaine 20 mg + ketamine 40 mg (*n* = 10). The mean difference in VAS score between inclusion and D56 was −25.60 (95% CI: −42.81, −8.39) in the lidocaine 20 mg group, −10.13 (95% CI: −26.24, 5.99) in the lidocaine 20 mg + ketamine 20 mg group, and −19.06 (95% CI: −36.11, −2.02) in the lidocaine 20 mg + ketamine 40 mg group. There was no statistically significant difference in change of VAS score between the 3 groups (*p* = 0.36). No infectious or severe adverse events occurred.

**Conclusion:**

This study did not demonstrate the superiority of mesotherapy with ketamine and lidocaine mixtures compared to lidocaine alone in relieving pain for CRPS1 patients. Lidocaine alone or mixed with ketamine can be administered safely with a rigorous mesotherapy procedure.

**Trial Registration:** The study was registered with ClinicalTrials.gov, number NCT04650074, and EudraCT, number 2020‐005557‐24

## 1 Introduction

Complex regional pain syndrome (CRPS) is characterized by a continuous, spontaneous or provoked regional pain, appearing disproportionate in intensity or duration compared to the expected evolution of the triggering event. This regional pain does not correspond to a peripheral nerve territory. It may be associated with motor, sensory, self‐motor, vasomotor, and trophic clinical signs. CRPS type 1 (CRPS1) occurs after a nociceptive traumatic event, while CRPS type 2 follows a proven nerve injury [1].

The diagnosis of CRPS is clinical, based on the diagnostic criteria from the International Association for the Study of Pain (IASP), known as the Budapest criteria [2]. The most frequent cause is trauma (40%). In 5% to 10% of cases, no triggering factor is found. The upper limb is more often affected. The pathophysiology of CRPS is complex and still insufficiently understood. Therefore, treatment of CRPS is symptomatic rather than curative, and multidimensional and multidisciplinary.

CRPS treatment is mainly directed towards the relief of symptoms. Various treatment modalities are available for managing these symptoms. Initial modalities comprise drugs and non‐invasive treatments such as physical therapy. In more severe forms of the disease with persistent symptoms, invasive treatments such as dorsal root ganglion stimulation may be offered although evidence for the efficacy of most CRPS interventions is weak [1, 3].

The use of ketamine in CRPS arouses interest without proof of its effectiveness [4, 5]. Ketamine is a fast‐acting anesthetic, which can produce an analgesic effect at low (subanesthetic) doses [6]. Ketamine has been studied as an adjuvant in the treatment of chronic pain, particularly neuropathic pain. Two literature reviews have synthesized results of studies assessing ketamine in CRPS using different routes of administration (IV, topical, oral) and doses, and concluded that there was not sufficient evidence supporting the efficacy of ketamine in this indication [4, 7].

Mesotherapy is a minimally invasive method that uses the properties of epidermal, dermal, or hypodermal tissue to obtain a specific action with small quantities of active substances [8]. It involves injecting low doses of medication into the skin, as close as possible to the area to be treated, for greater efficacy and to limit systemic side effects. Injected solutions often contain a local anesthetic such as lidocaine for better local tolerance. Two reviews have evaluated the analgesic efficacy of mesotherapy in the treatment of localized pain [8, 9]. Mesotherapy has been shown to be especially promising for calcified tendinitis of the shoulder [10] and in the rehabilitation treatment of musculoskeletal pain [9].

In this randomized trial, we assessed for the first time the efficacy of ketamine and lidocaine mixtures versus lidocaine alone administered by mesotherapy in CRPS1, in addition to standard treatment. We hypothesized that the combination of ketamine and lidocaine was superior to lidocaine alone on pain relief.

## 2 Methods

### 2.1 Study Design

The study was a 3‐arm, double‐blind, randomized trial comparing mesotherapy with lidocaine 20 mg alone or mixed with ketamine at a dose of 20 or 40 mg. It was conducted in the lower limb orthopedic surgery department of the Hospices Civils de Lyon (Lyon, France). The protocol was approved by an ethics committee (Comité de Protection des Personnes Ile de France VII, decision SI 21.02171.021013), and all included patients provided written informed consent. The study was registered with ClinicalTrials.gov, number NCT04650074, and EudraCT, number 2020‐005557‐24.

### 2.2 Participants

Inclusion criteria were as follows: patients over 18 years old, with a diagnosis of CRPS1 of the upper or lower limb according to the Budapest criteria [2] with a neuropathic component diagnosed using the DN4 questionnaire (*Douleur Neuropathique 4 Questions*) [11], having undergone a dynamic 3‐phase bone scan (vascular, tissue, bone) within the last 3 months showing diffuse and extensive hyperfixation in the suspected CRPS1 area, and having a visual analog scale (VAS) > 50 mm.

Criteria for non‐inclusion included: medical histories or current pathologies (epilepsy, hypertension, unbalanced coronary artery disease, myocardial infarction within the last 12 months, porphyria, hyperthyroidism, known Behçet's disease, known blood coagulation disorder or prothrombin level < 20%, known psychiatric disorders, known septic osteo‐articular pathology), HIV infection, immunodeficiency and/or immunosuppressive treatment, history of severe allergy (Quincke's edema), severe heart failure, known allergies to chromium and/or nickel, ongoing skin infection, skin lesion near the injection area, injection phobia, known hypersensitivity to ketamine hydrochloride or chlorobutanol, known hypersensitivity to lidocaine hydrochloride or amide blonded local anesthetics, pregnancy or breastfeeding.

### 2.3 Randomization and Blinding

After obtaining written consent, a urine pregnancy test was performed for women of childbearing age. All participants completed questionnaires for smoking (Fagerström Test for Nicotine Dependence, FTND), alcohol use (Alcohol Use Disorders Identification Test, AUDIT), and drug/substance use (Drug Abuse Screening Test, DAST‐20). If the pregnancy test and the results of the questionnaires met the eligibility criteria (FTND score ≤ 8, AUDIT score ≤ 12 in men and ≤ 11 in women, and DAST‐20 score ≤ 11), the patient was included in the study and randomized in a 1:1:1 ratio.

Randomization was carried out at individual level in blocks of variable size, without stratification. Treatment allocation was computer‐generated by the Hospices Civils de Lyon's clinical trial unit (Lyon, France), using an IWRS (Ennov Clinical V8.2.20.4l), and was double‐blind (participant, physician). A notification with allocated treatment to dispense was sent by email to the clinical trial pharmacist before each mesotherapy session.

Outcomes were assessed by a clinical research assistant who did not know the group the patient had been assigned.

### 2.4 Treatment Procedures

The investigational medicinal products (IMPs) used in this study consisted of sterile injectable solutions prepared, quality‐controlled, and supplied by the pharmacy of Edouard Herriot Hospital (FRIpharm, Hospices Civils de Lyon, France). IMPs were numbered and were allocated according to patient randomization into three treatment groups: (1) an injectable solution of lidocaine hydrochloride 20 mg (3.33 mg/mL) in the “lidocaine 20 mg” group; (2) an injectable solution combining lidocaine hydrochloride 20 mg (3.33 mg/mL) and ketamine 20 mg (3.33 mg/mL) in the “lidocaine 20 mg + ketamine 20 mg” group; (3) an injectable solution combining lidocaine hydrochloride 20 mg (3.33 mg/mL) and ketamine 40 mg (6.67 mg/mL) in the “lidocaine 20 mg + ketamine 40 mg” group.

All IMPs were presented in same packaging (in terms of color, size, and appearance) to ensure treatment blinding. Preparation was performed in compliance with the Good Manufacturing Practices (GMP) defined by the French National Agency for Medicines and Health Products Safety (ANSM, 2007 version). The solutions were prepared from commercially available injectable drug products: Lidocaine Aguettant 10 mg/mL solution for injection (AGUETTANT, France), Kétamine Renaudin 50 mg/mL solution for injection (RENAUDIN, France), and 0.9% sodium chloride solution for injection (MACOPHARMA, France). All IMPs were filled into 10‐mL type I clear glass vials, with 6 mL of solution per vial, and sealed with perfluorinated bromobutyl stoppers crimped with aluminum caps. The preparation and quality control of these sterile injectable solutions were carried out in accordance with the requirements of the European Pharmacopeia for intradermal injectable preparations, including specifications on solution clarity, pH, sterility, and endotoxin levels.

A stability study, conducted according to the International Council for Harmonization (ICH) guidelines on stability testing (ICH Q1A[R2]), demonstrated that all formulations remained chemically and physically stable for at least 18 months at room temperature, covering the full duration of the study.

A total of 4 mesotherapy sessions were planned per patient (on D1, D7, D14, and D28). During each mesotherapy session, a trained physician injected 6 mL of the blinded solution (lidocaine hydrochloride 20 mg alone or combined with ketamine 20 mg or ketamine 40 mg).

Each mesotherapy session included an intra‐epidermal and intra‐dermal injection, following 5 steps [12]: (1) achieving asepsis on the treated area with alcoholic Chlorhexidine 2% (or Betadine dermal or Dakin), (2) air‐drying and taking 6 mL of the injectable solution from the numbered vial assigned to the patient for the visit, (3) achieving a second asepsis with the same product as in step 1, (4) performing the first intra‐epidermal sequence by crossed lines with manual technique using a 13 mm × 0.30 needle (injection of 3 mL of the solution), (5) carrying out the second intra‐dermal sequence at 1 mm with Pistor Eliance injector assisted technique at a frequency of 200 punctures per minute (injection of the remaining 3 mL of the solution). In the end, all the solution was injected to the patient. After injection, patients were monitored for 15 min with blood pressure measurements. Total duration of a session was about 30 min.

### 2.5 Standard Treatment

In all groups, participants could benefit from usual management of pain, such as analgesic drugs or non‐invasive treatments (physical therapy, occupational therapy…).

### 2.6 Outcomes

The primary outcome was the change in pain between inclusion and Day 56 after 4 mesotherapy sessions, assessed by a visual analog scale (VAS). Pain was rated on a scale from 0 to 100 mm; the higher the score, the more intense the pain.

The pain secondary outcomes were the evolution of pain using different scales: the VAS score, the Neuropathic Pain Symptom Inventory (NPSI), and the Brief Pain Inventory (BPI).

NPSI is a self‐questionnaire that includes 12 items: 10 descriptors of the different symptoms and 2 items for assessing the duration of spontaneous ongoing and paroxysmal pain. The tool evaluates mean pain intensity in the last 24 h in a verbal numeric scale from zero (no pain) to 10 (worst imaginable pain). Total intensity pain score is calculated by the sum of 10 descriptors. A score is also calculated for five dimensions of neuropathic pain [13]: superficial burning pain, deep pain, paroxysmal pain, paraesthesia/dysesthesia, and allodynia/hyperalgesia.

The short version of the BPI self‐questionnaire was used to evaluate the pain intensity (severity) assessed with 4 items, and the impact of pain on functioning (interference) assessed with 7 items [14]. Both BPI scales yield 0 to 10 scores (higher score = worse function or intensity).

As secondary outcomes, the European Quality of Life 5 Dimensions 3 Level questionnaire (EQ‐5D‐3L) was used to assess the quality of life. The EQ‐5D‐3L is a self‐questionnaire comprising five dimensions: mobility, self‐care, usual activities, pain/discomfort, and anxiety/depression. For each dimension, there are three levels of responses: “no problems,” “some problems,” and “extreme problems.” A total score is then calculated, with a score of 1 corresponding to the best possible health state, and a value below 0 represents the worst possible health state [15].

VAS and NPSI were measured at inclusion (D0), before injection at each mesotherapy session (D1, D7, D14, D28), and at the end‐of‐study visit (D56), while BPI and EQ‐5D‐3L were only measured at inclusion and D56.

Adverse events and use of concomitant analgesic treatments were collected throughout the study. Adverse events were graded according to the Common Terminology Criteria for Adverse Events (CTCAE version 5.0).

### 2.7 Statistical Analysis

The inclusion of at least 30 patients in total (10 per group) allowed for a power of 83.3% to conclude a significant difference between groups on the VAS score change at Day 56 since inclusion. The hypotheses involved an expected VAS score of 80 mm (SD = 10) at inclusion for all three groups, and at Day 56, of 40 mm (SD = 10) in the lidocaine group, 30 mm (SD = 10) in the lidocaine + ketamine 20 mg group, and 20 mm (SD = 10) in the lidocaine + ketamine 40 mg group. Under these assumptions and considering 15% loss of follow‐up, up to 12 patients per group were allowed to detect a difference between groups using a trend test based on simulations with Beta distributions for the VAS score in each group at inclusion and at Day 56.

All analyses were performed in the intention‐to‐treat population. Qualitative and quantitative baseline characteristics were described in each group using respectively the number and the percentage in each category, or the median and the interquartile range.

The change in the VAS score was analyzed using a linear regression model including VAS score at inclusion (centered to mean) and treatment group. A likelihood ratio test and a trend test (if a linear trend of the group effect was observed) were used for the comparison between the three groups. The same analysis was performed to compare the change at Day 56 since inclusion on BPI scores and on EQ‐5D‐3L score between groups.

To take into account the longitudinal measurement (at inclusion, Day 1, Day 7, Day 14, Day 28, and Day 56), the evolution of the VAS score was also compared between groups using a linear mixed model. The model included patients as a random effect (on the intercept and the slope) and as fixed effects: time since inclusion, treatment group (with the lidocaine group as the reference), and an interaction between the group and time. The same analysis was carried out to compare the evolution of NPSI score between groups. Adverse events occurring during the study were described by treatment groups.

Statistical significance was defined by a two‐sided *p* < 0.05. All analyses were performed using R, version 4.4.0.

## 3 Results

**FIGURE 1:**
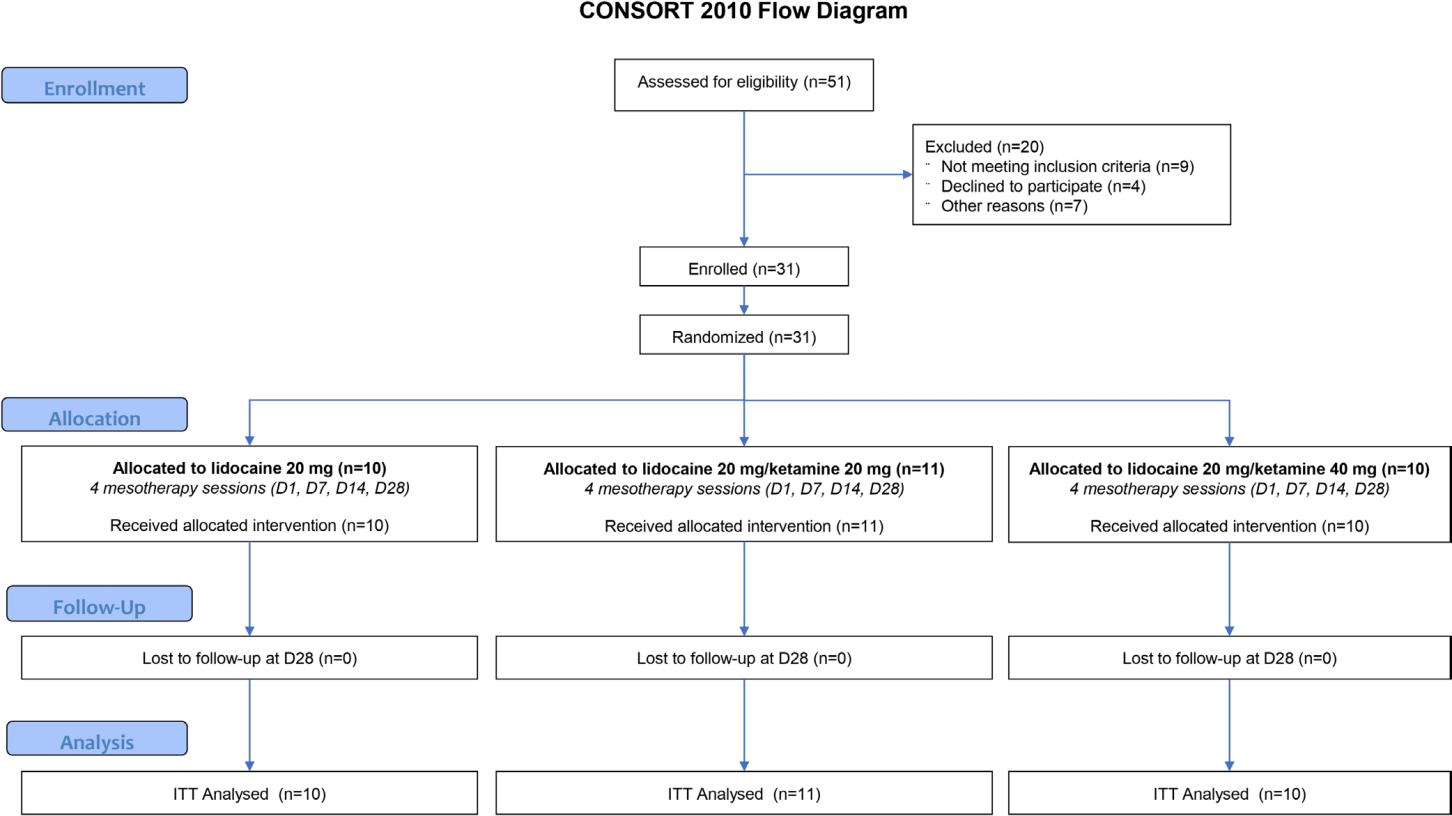
Flowchart of the trial.

Thirty‐one patients were recruited between November 2021 and November 2023: 10 in the lidocaine 20 mg group, 11 in the lidocaine 20 mg + ketamine 20 mg group, and 10 in the lidocaine 20 mg + ketamine 40 mg group. The flowchart is shown in Figure 1. Participant characteristics are presented in Table 1. Overall, there was a majority of women with CRPS1 of the lower limbs.

**TABLE 1:** Characteristics of participants.

	Lidocaine 20 mg (*N* = 10)	Lidocaine 20 mg + ketamine 20 mg (*N* = 11)	Lidocaine 20 mg + ketamine 40 mg (*N* = 10)
Age (year)	37.00 (29.25–42.75)	48.00 (37.00–54.50)	53.50 (45.75–55.75)
Female	5 (50%)	8 (72.7%)	6 (60%)
Disease duration since CRPS1 diagnosis (days)	52.00 (20.00–90.00)	70.00 (38.00–132.00)	82.50 (8.00–148.00)
Limbs affected			
Lower limbs	7 (70%)	10 (90.9%)	10 (100.0%)
Upper limbs	3 (30.0%)	1 (9.1%)	0
Neuropathic pain (DN4 score)	7.00 (6.25–8.00)	7.00 (6.50–8.00)	7.00 (5.25–8.00)

*Note:* Values are median (Q1–Q3) for quantitative variables or effective (percentage) for qualitative variables.

### 3.1 Primary Outcome

Mean difference in VAS score between inclusion and D56 based on the adjusted regression linear model was −25.6 (95% CI: −42.8, −8.4) in the lidocaine 20 mg group, −10.1 (95% CI: −26.2, 6.0) in the lidocaine 20 mg + ketamine 20 mg group, and −19.1 (95% CI: −36.1, −2.0) in the lidocaine 20 mg + ketamine 40 mg group (Table 2). There was no statistically significant difference in mean change of VAS score between the three groups (*p* = 0.36). The estimated difference in change of VAS score from inclusion to D56 was 15.47 points (95% CI −8.24 to 39.18) between the lidocaine 20 mg + ketamine 20 mg group and the lidocaine 20 mg group (reference group), in favor of the lidocaine 20 mg group. It was 6.53 points (95% CI −18.02 to 31.09) between the lidocaine 20 mg + ketamine 40 mg group and the lidocaine 20 mg group, in favor of the lidocaine 20 mg group.

**TABLE 2:** Primary and secondary outcomes at inclusion and D56 (Intention‐to‐treat population).

Outcome	Treatment group	At inclusion, mean (SD)	At D56, mean (SD)	Mean difference (95% CI)^a^	*p* ^b^
VAS	Lidocaine 20 mg	75.30 (8.83)	47.20 (31.30)	−25.60 [−42.81, −8.39]	0.36
	Lidocaine 20 mg/ketamine 20 mg	70.91 (11.74)	61.45 (19.91)	−10.13 [−26.24, 5.99]	
	Lidocaine 20 mg/ketamine 40 mg	69.40 (10.02)	52.10 (25.20)	−19.06 [−36.11, −2.02]	
BPI severity	Lidocaine 20 mg	6.55 (1.04)	4.65 (2.20)	−1.69 [−2.96, −0.43]	
	Lidocaine 20 mg/ketamine 20 mg	6.27 (1.21)	6.11 (1.15)	−0.07 [−1.26, 1.12]	
	Lidocaine 20 mg/ketamine 40 mg	5.35 (2.24)	5.12 (2.70)	−0.53 [−1.82, 0.76]	
BPI interference	Lidocaine 20 mg	5.64 (1.42)	4.06 (2.30)	−1.59 [−2.92, −0.26]	
	Lidocaine 20 mg/ketamine 20 mg	5.97 (1.79)	5.57 (1.90)	−0.20 [−1.48, 1.07]	
	Lidocaine 20 mg/ketamine 40 mg	5.31 (2.41)	5.07 (2.22)	−0.45 [−1.79, 0.88]	
EQ‐5D‐3L	Lidocaine 20 mg	0.32 (0.23)	0.47 (0.14)	0.15 [0.02, 0.29]	
	Lidocaine 20 mg/ketamine 20 mg	0.34 (0.26)	0.29 (0.27)	−0.03 [−0.17, 0.10]	
	Lidocaine 20 mg/ketamine 40 mg	0.31 (0.26)	0.37 (0.22)	0.05 [−0.09, 0.19]	

*Note:* Score interpretation: higher scores on the VAS and BPI scales indicate greater pain; lower scores on the EQ‐5D‐3L indicate lower quality of life.

^a^ Mean difference and 95% confidence interval calculated from linear regression model adjusted on the inclusion value (centered on mean value).

^b^ *p*‐value of the likelihood ratio test for global treatment effect.

### 3.2 Secondary Outcomes

Scores of severity and interference of the BPI and scores of quality of life at inclusion and D56 are reported in Table 2.

The difference in the change of severity score from inclusion to D56 was 1.62 points (95% CI −0.10 to 3.35) and of interference score was 1.39 points (95% CI −0.45 to 3.23) between the lidocaine 20 mg + ketamine 20 mg group and the lidocaine 20 mg group (in favor of the lidocaine 20 mg group). Similarly, the difference in the change of severity score was 1.17 points (95% CI −0.68 to 3.01) and of interference score was 1.14 points (95% CI −0.75 to 3.02) between the lidocaine 20 mg + ketamine 40 mg and the lidocaine 20 mg group (in favor of the lidocaine 20 mg group).

As compared with the lidocaine 20 mg group (reference group), the change in quality of life score from inclusion to D56 differed on average by −0.19 points (95% CI −0.38 to 0.00) for the lidocaine 20 mg + ketamine 20 mg, and by −0.10 points (95% CI −0.30 to 0.09) for the lidocaine 20 mg + ketamine 40 mg group, indicating a more pronounced increase in the lidocaine 20 mg group.

**FIGURE 2:**
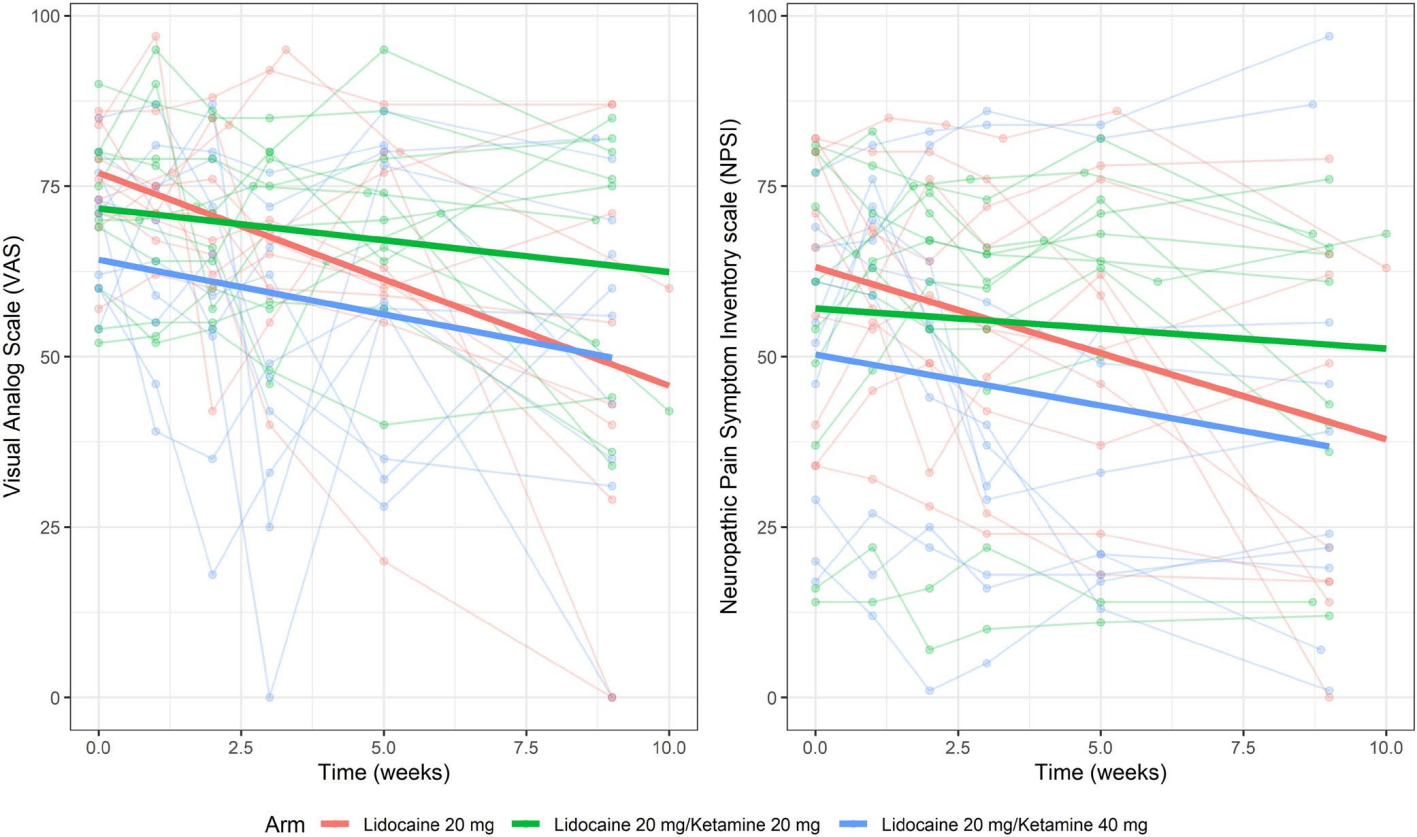
Evolution of Visual Analog Scale (VAS) and Neuropathic Pain Symptom Inventory (NPSI) scores.

Evolution of VAS and NPSI scores over time are presented in Table 3 and Figure 2. At inclusion, VAS score was significantly lower in the lidocaine 20 mg + ketamine 40 mg group compared to the lidocaine 20 mg group. As compared with the lidocaine 20 mg group, the difference in change of VAS score was 2.19 (95% CI −0.20 to 4.58) for the lidocaine 20 mg + ketamine 20 mg group and 1.53 (95% CI −0.92 to 3.97) for the lidocaine 20 mg + ketamine 40 mg group, indicating a more pronounced decline in the lidocaine 20 mg compared to the other groups. In the same way for NPSI, compared to the lidocaine 20 mg group (reference group), the difference in change of NPSI score was 1.94 (95% CI −0.34 to 4.21) for the lidocaine 20 mg + ketamine 20 mg group and 1.03 (95% CI −1.30 to 3.36) for the lidocaine 20 mg + ketamine 40 mg group.

**TABLE 3:** Secondary outcomes: Evolution of the VAS and NPSI scores.

Outcome	Treatment group	Mean difference at inclusion vs. reference group^a^	Mean difference of change per week vs. reference group^a^
VAS	Lidocaine 20 mg^b^	76.95 [70.07, 83.83]	−3.12 [−4.85, −1.40]
	Lidocaine 20 mg/ketamine 20 mg	−5.20 [−15.07, 4.66]	2.19 [−0.20, 4.58]
	Lidocaine 20 mg/ketamine 40 mg	−12.76 [−22.85, −2.67]	1.53 [−0.92, 3.97]
NPSI	Lidocaine 20 mg^b^	63.14 [49.47, 76.81]	−2.53 [−4.17, −0.88]
	Lidocaine 20 mg/ketamine 20 mg	−6.07 [−25.66, 13.52]	1.94 [−0.34, 4.21]
	Lidocaine 20 mg/ketamine 40 mg	−12.82 [−32.86, 7.23]	1.03 [−1.30, 3.36]

*Note:* Score interpretation: higher scores on the VAS and NPSI scales indicate greater pain.

^a^ Mean difference and 95% confidence interval calculated from linear mixed model.

^b^ Reference group.

The majority of patients reported any consumption of analgesics during the study (24 out of 31 patients).

Six patients reported mild adverse events (i.e., grade 1) during the study (Table 4). In the lidocaine 20 mg group, one patient had dermatological adverse event and pain at injection site, and one patient experienced headache with drowsiness. In the lidocaine 20 mg + ketamine 20 mg group, one patient had a dermatological adverse event and one patient described headache. In the lidocaine 20 mg + ketamine 40 mg group, one patient presented a dermatological adverse event, and one patient reported pain at injection site and drowsiness. No serious adverse events nor infectious adverse events were reported during the study. All adverse effects were temporary and resolved spontaneously or quickly with appropriate treatment.

**TABLE 4:** Adverse events (AE).

	Lidocaine 20 mg	Lidocaine 20 mg + ketamine 20 mg	Lidocaine 20 mg + ketamine 40 mg
	(*N* = 10)	(*N* = 11)	(*N* = 10)
At least one AE	2 (20.0%)	2 (18.2%)	2 (20.0%)
Type of AE^a^			
AE dermatologic	1 (10.0%)	1 (9.1%)	1 (10.0%)
Pain at injection site	1 (10.0%)	0	1 (10.0%)
Drowsiness	1 (10.0%)	0	1 (10.0%)
Headache	1 (10.0%)	1 (9.1%)	0

^a^ A total of 6 patients reported 16 AE. The same patient could report the same type of AE several times.

## 4 Discussion

This clinical trial assessed for the first time the effects of mesotherapy in CRPS1. As there is no consensus on the treatment of patients suffering from CRPS1 and there are strong arguments to evaluate mesotherapy in this context, we proposed a randomized trial intended to evaluate the effects on disease symptoms and safety of different mesotherapy dosages and mixtures.

The results did not reveal statistically significant difference between the three mesotherapy groups for changes in VAS pain score between inclusion and D56. Changes in pain with other pain scales and in quality of life were similar between the three groups. Although the study doesn't allow conclusion due to wide confidence intervals, the lidocaine 20 mg group generally seemed to have better results, while the lidocaine 20 mg + ketamine 20 mg group seemed to have the worst outcomes. The safety assessment did not highlight any risk to the patient. Adverse events were rare and transitory, such as erythema or pain at the injection site, headache, and drowsiness.

CRPS1 is a disabling syndrome whose primary therapeutic objectives are analgesia, but also maintaining or increasing joint amplitude, and maintaining or restoring motor function. The therapeutic strategy must be graduated and adapted. First‐line treatment combines analgesics adapted to the pain and early rehabilitation. Passive medical devices such as orthoses or canes can also be used for analgesic or functional preservation purposes. If the evolution is more severe, with intense pain and functional repercussions, multidisciplinary care involving specific treatment may be proposed, including: drugs (e.g., opioid analgesic agents, oral steroids, bisphosphonates, ketamine), non‐invasive interventions (e.g., supportive psychotherapy, physical therapy) and invasive interventions (e.g., sympathetic blocks, spinal cord stimulation, dorsal root ganglion stimulation) [1, 3, 5, 16, 17]. However, there is no consensus on the overall treatment strategy.

Mesotherapy is a minimally invasive and easy to apply method. Numerous studies have assessed its interest for some conditions [8, 9]. Based on the scientific literature, there was a rationale to study the analgesic efficacy of mesotherapy with lidocaine and ketamine in CRPS1 patients. In our study, the injected solutions contained 20 mg of lidocaine alone or combined with ketamine. All solutions of lidocaine and ketamine could be injected intraepidermally and intradermally without complications during each session, for a total of 4 sessions 1 week apart.

In a literature review evaluating mesotherapy in the rehabilitation treatment of musculoskeletal pain [9], some studies used a lidocaine‐based solution with similar doses of lidocaine (i.e., between 10 and 40 mg). Recently, a randomized trial comparing intradermal mesotherapy with systemic therapy in the treatment of low back pain used lidocaine at a dose of 16 mg in mixture [18]. Overall, these studies reported only minor and non‐infectious side effects of mesotherapy, which is consistent with our results.

Some studies used ketamine in the treatment of CRPS, but evidence of its efficacy is still lacking [4]. Moreover, no studies have investigated ketamine in mesotherapy. In the absence of recommendations on the use of ketamine in mesotherapy, ketamine doses were based on those used intravenously. Intravenous ketamine is known to have an analgesic effect at subanesthetic doses of 0.15 to 0.25 mg/kg [6], and common subanesthetic doses used in clinical practice are of 0.2 to 0.9 mg/kg/day [19, 20, 21]. In our study, we used corresponding doses of 20 to 40 mg of ketamine per injection, combined with 20 mg of lidocaine.

Lidocaine is a short‐acting amide local anesthetic, which can block sodium channels and inhibit N‐methyl‐D‐aspartate (NMDA) receptors, all being potentially involved in pain pathways [22]. In general, lidocaine is mixed with other products such as nonsteroidal anti‐inflammatory drugs, muscle relaxants, anti‐edematous drugs, or calcitonin [12]. The choice of the mixture depends on the physician's experience rather than experimental data.

Ketamine is a fast‐acting non‐barbiturate anesthetic at high doses and an analgesic at low doses. It is mainly distributed in richly vascularized organs. It is a short‐acting, highly‐lipid soluble molecule. It belongs to the family of NMDA receptor antagonists [23]. Lidocaine and ketamine share several common effects through different mechanisms [22, 23, 24]. They both block sodium channels and inhibit NMDA receptors, probably by interacting at different sites of these receptors [24]. Research has shown that NMDA receptors amplify the activation of spinal nociceptive neurons involved in the induction and maintenance of central sensitization and thus play a role in the development and chronicity of pain [25]. All together, these elements suggest that their association could potentiate their effects. However, our study did not show a greater analgesic effect of the combination compared to lidocaine alone.

The main strength of this study was the well‐conducted randomized trial, with double‐blind assessment of the primary outcome measure. All included patients were evaluated for the primary outcome and analyzed on an intention‐to‐treat basis. To the best of our knowledge, this study is the first to evaluate mesotherapy in patients with CRPS1. Moreover, all mesotherapy sessions were performed by an experienced physician, trained in mesotherapy, following a rigorous process. Compatibility and stability of the ketamine and lidocaine mixtures were evaluated before the start of the study, and the safety of the treatment procedure was rigorously assessed throughout the study.

The study also has some limitations. This was a single‐center study on a relatively small sample of patients, explaining baseline imbalances between groups in particular for pain level at inclusion. The sample size of the study was based on optimistic assumptions regarding the expected effect size. Consequently, the study was underpowered to detect a lower effect size that can be clinically meaningful. Moreover, in all 3 mesotherapy groups, injected solutions contained active substances so as to determine whether a solution was worth investigating. There was no comparison to a placebo group (e.g., mesotherapy with physiological saline injection). Therefore, a specific effect of the active substances cannot be distinguished from a placebo effect of injections, particularly in the lidocaine 20 mg group. The placebo effect in pain syndromes has been widely studied, and is more marked for invasive treatments [26]. In a study comparing 3 interventions for the treatment of tendinitis (mesotherapy with nonsteroidal anti‐inflammatory drug injection or physiological saline injection or dry injection), pain symptoms improved in all groups, suggesting an important effect of the injection in mesotherapy [27].

## 5 Conclusions

Our study did not permit demonstrating the efficacy of a mixture of ketamine and lidocaine compared to lidocaine alone administered by mesotherapy in the context of CRPS1. However, lidocaine alone or mixed with ketamine can be administered safely with a rigorous injection technique. Larger‐scale clinical trials with a placebo group are encouraged to determine the specific benefits of mesotherapy as an additional treatment in the overall treatment strategy of CRPS1.

## Author Contributions

P.L., L.M., J.W., C.B., J.H., F.R., C.D., and O.F.‐C. contributed to the conception and design. P.L. and M.K. contributed to the acquisition of data. A.A.‐K. and M.R. analyzed the data. All authors contributed to the interpretation of the results, commented on the manuscript, and approved the final version of the manuscript.

## Funding

The study was funded by the French Society of Mesotherapy, the APICIL Foundation, the Health Mutual MCLR69, the Hospices Civils de Lyon Foundation, and MI Medical Innovation (grant and Pistor Eliance equipment).

## Ethics Statement

The protocol was approved by an ethics committee (Comité de Protection des Personnes Ile de France VII, decision SI 21.02171.021013).

## Consent

All included patients provided written informed consent.

## Conflicts of Interest

The authors declare no conflicts of interest.

## Data Availability

Data generated during the current study is available from the corresponding author on reasonable request.
